# Supplementary material for: A mechanical-assisted post-bioprinting strategy for challenging bone defects repair
Source: Nat Commun. 2024 Apr 26;15:3565. doi: 10.1038/s41467-024-48023-8 (PMC11053166; doi:10.1038/s41467-024-48023-8)
Supplement: Supplementary file 1 — Supplementary Information [file 41467_2024_48023_MOESM1_ESM.pdf]

Supplementary Information

A Mechanical-assisted Post-bioprinting Strategy for Challenging Bone Defects Repair

*Jirong Yang[1,2], Zhigang Chen[1,2], Chongjian Gao[1], Juan Liu[1], Kaizheng Liu[1], Xiao Wang[1,3], Xiaoling Pan[1,3], Guocheng Wang[1,2], Hongxun Sang[3], Haobo Pan[1,2], Wenguang Liu[4], Changshun Ruan[1,2,5] **

[1] Research Center for Human Tissue and Organ Degeneration, Institute of Biomedicine and Biotechnology, Shenzhen Institute of Advanced Technology, Chinese Academy of Sciences, Shenzhen 518055, China

[2] University of Chinese Academy of Sciences, Beijing 100049, China

[3] Shenzhen Hospital, Southern Medical University, Shenzhen 518000, PR China

[4] School of Materials Science and Engineering, Tianjin Key Laboratory of Composite and Functional Materials, Tianjin University, Tianjin 300350, China

[5] The Key Laboratory of Biomedical Imaging Science and System, Chinese Academy of Sciences

*Corresponding author (E-mail: cs.ruan@siat.ac.cn)

Supplementary Figs

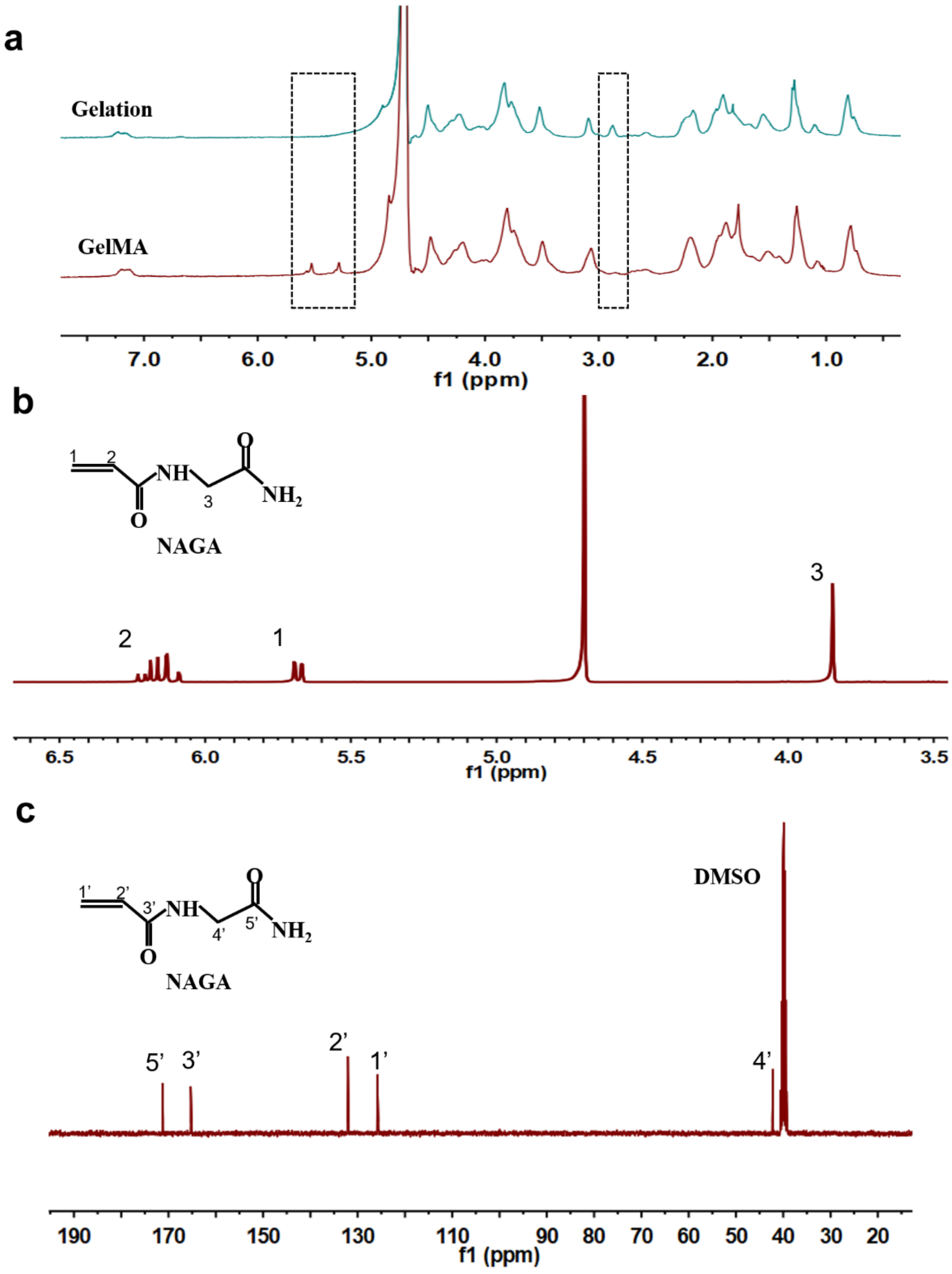

Supplementary Fig. 1 Characterization of the synthesized GelMA and NAGA. a, ^{1}H NMR spectrum of synthesized GelMA in D_2O. Two distinctive peaks at about δ 5.6 ppm and δ 5.4 that were attributed to the protons of double bond ($C = CH_2$) appeared, indicating successful grafting of MA in gelatin. The quantitative analysis of the ^{1}H NMR spectrum shows that the degree of methacrylate of gelatin was 67 ± 2%. **b**, ^{1}H NMR spectrum of NAGA in D_2O. **c**, ^{13}C NMR spectrum of NAGA in DMSO-d_6.

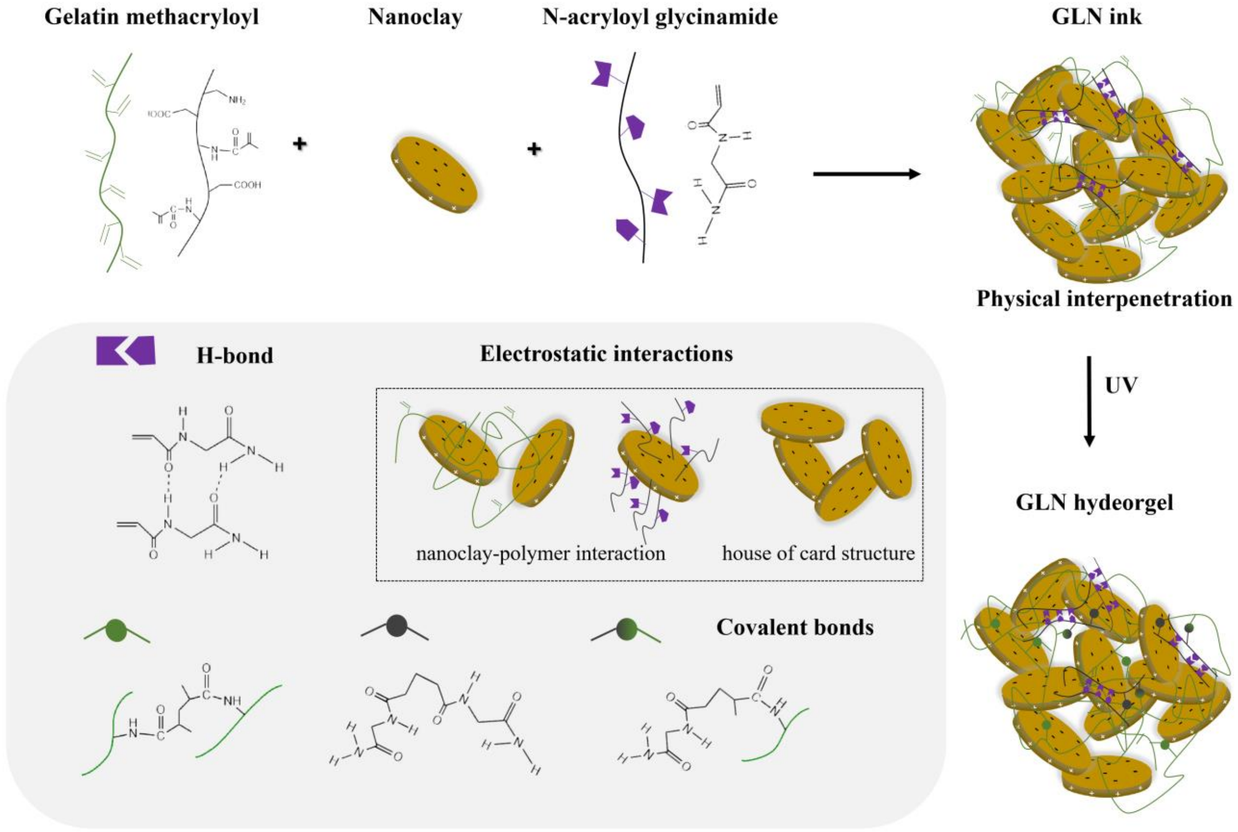

Supplementary Fig. 2 The schematic diagram of the physical interpenetration of GLN hybrid inks.

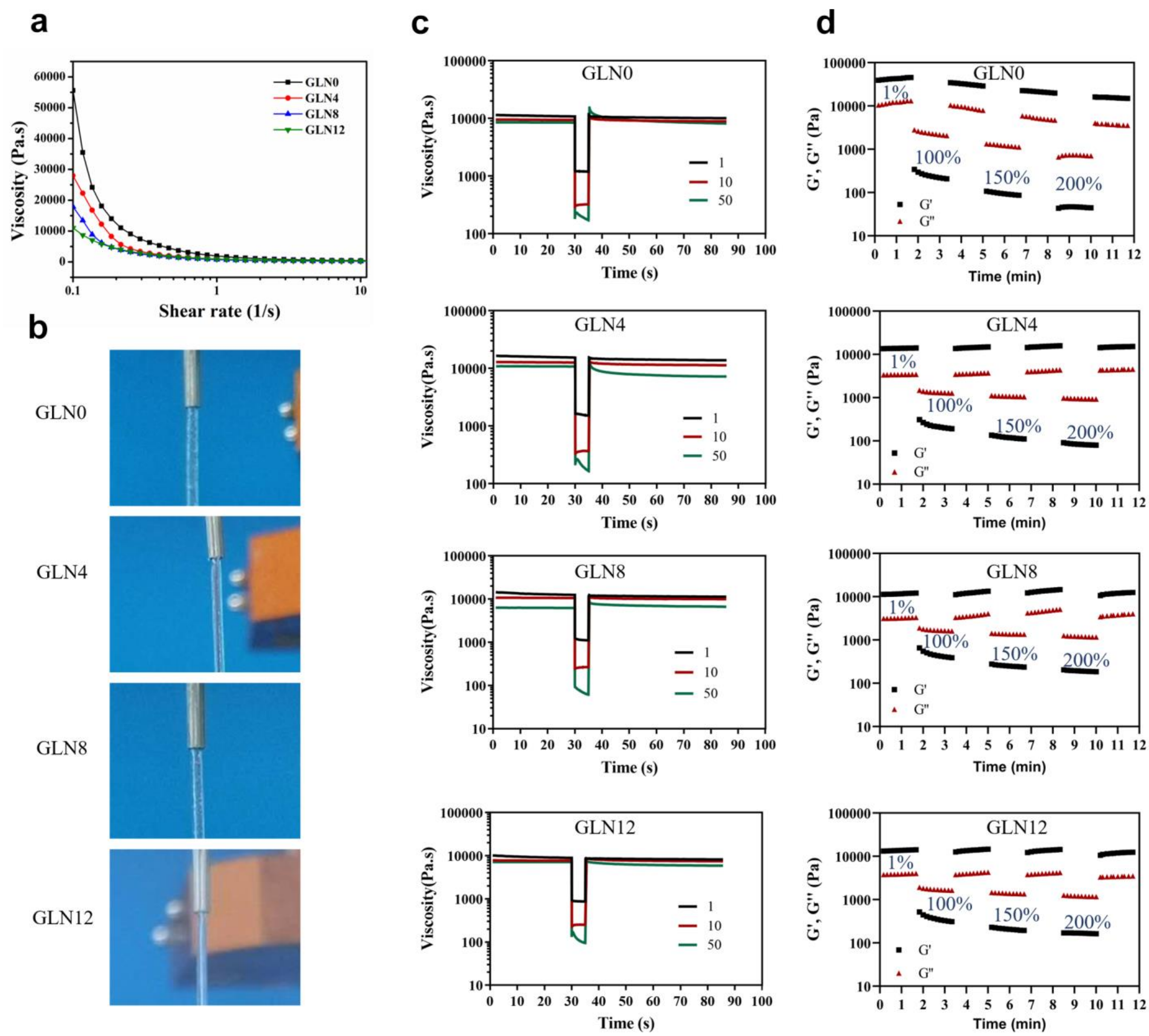

Supplementary Fig. 3 Rheological properties of GLN hybrid inks. a, Viscosity of hybrid GLN inks in a shear rate sweep from 0.1 to 10 s^{-1} at 25 °C. **b**, Photographs of extruded hollow filaments via a coaxial nozzle using GLN hybrid inks without supporting materials. **c**, Viscosity of GLN hybrid inks in shear rate sweeps at three stages with shear rate 0.1 s^{-1} (60 s) **-** 1 or 10 or 50 s^{-1} (5 s) **-** 0.1 s^{-1} (60 s) at 25 ºC. **d**, Storage modulus (G') and loss modulus (G'') of GLN hybrid inks in shear strain sweeps from successively strain 1% (90 s) to strain 50%, 100%, 150% (90 s), with f = 1 Hz and T = 25 ºC. Source data are provided as a Source Data file.

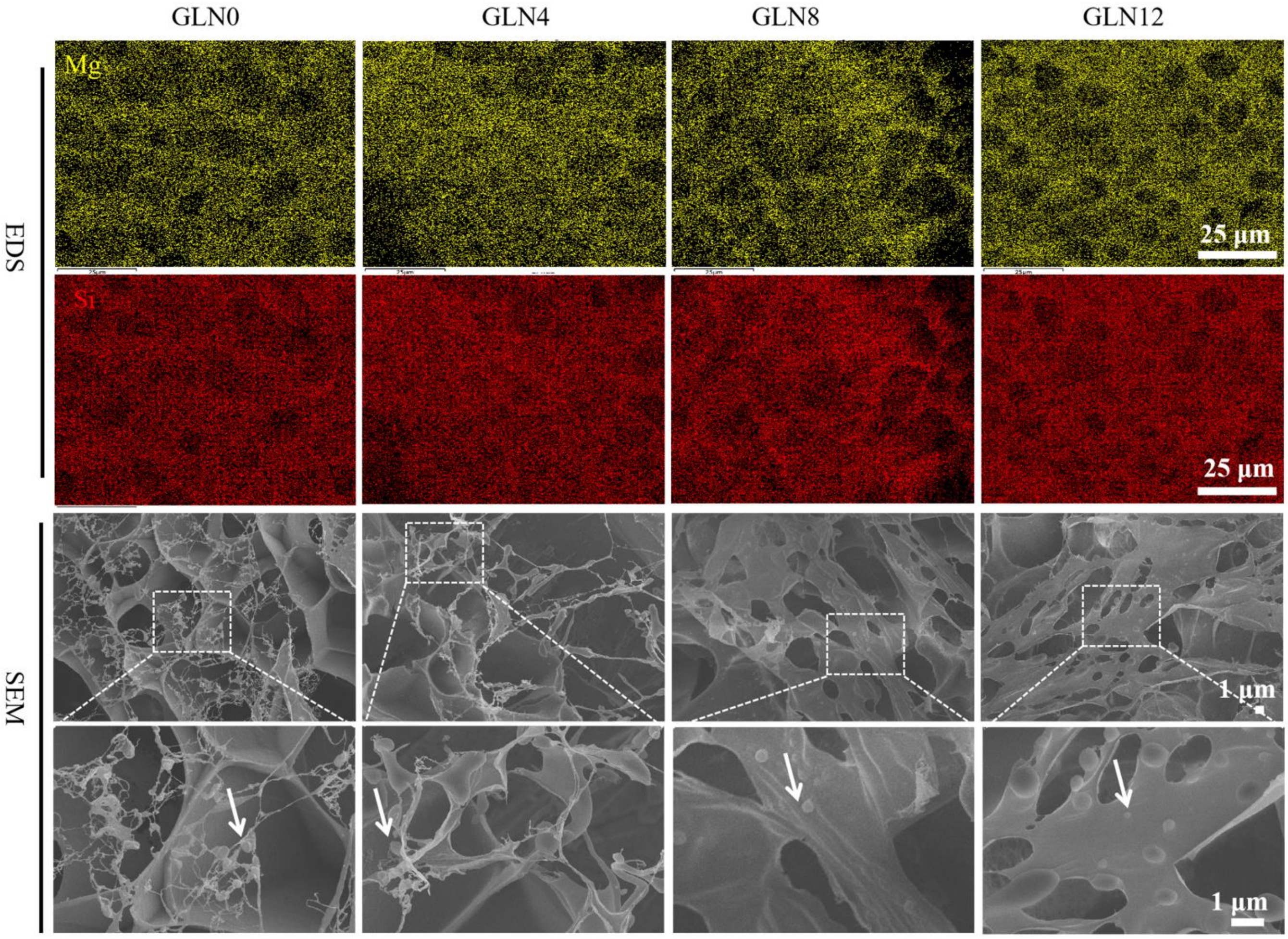

Supplementary Fig. 4 Microscopic morphology observation (EDS and SEM) of GLN hydrogels.

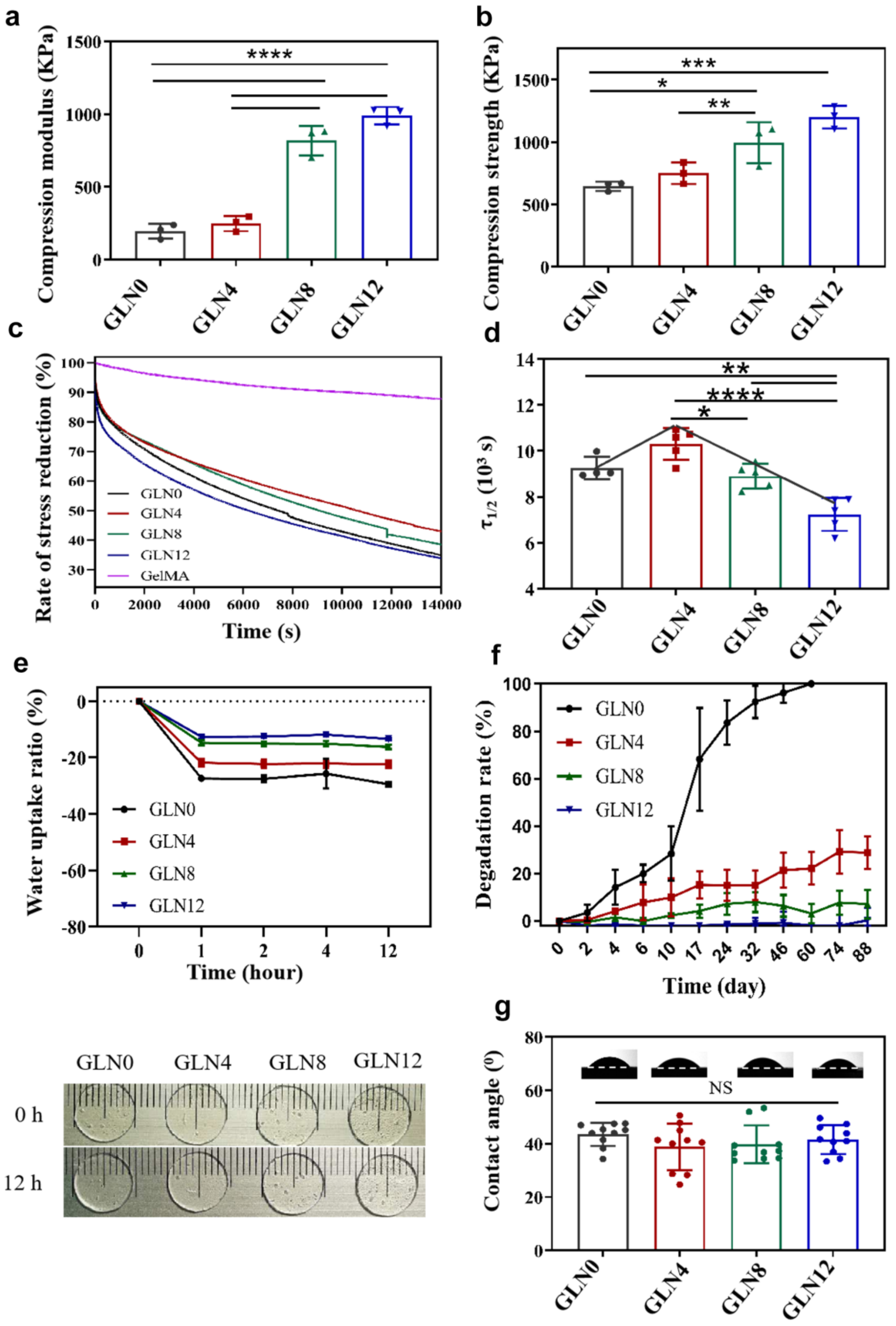

Supplementary Fig. 5 Physical properties of GLN hydrogels. a,**b**, Compression modulus, n = 3 per group (**a**) and compression strength, ***P < 0.0001 (**b**) of GLN hydrogels, *P = 0.0155, **P = 0.0035, ***P = 0.0009. **c**,**d**, The curve of stress relaxation (**c**) and relaxation time (**d**) defined as the time that the stress reduces to half of the initial stress under 20% strain, n = 5 per group, *P = 0.0138, **P = 0.0012 (GLN0 and GLN12), **P = 0.0037 (GLN8 and GLN12), ****P < 0.0001. **e**, Water uptake of GLN hydrogels in PBS at 37 ºC for 12 h. **f**, Degradation of GLN hydrogels in PBS at at 37 ºC, n = 4 per group. **g**, Water contact angle of GLN hydrogels, n = 10 per group, NS represents no significant difference (P > 0.05). Dates are presented as means ± s.d, statistical significance was calculated using one-way ANOVA method with Tukey's multiple comparisons tests, NS represents no significant difference. Source data are provided as a Source Data file.

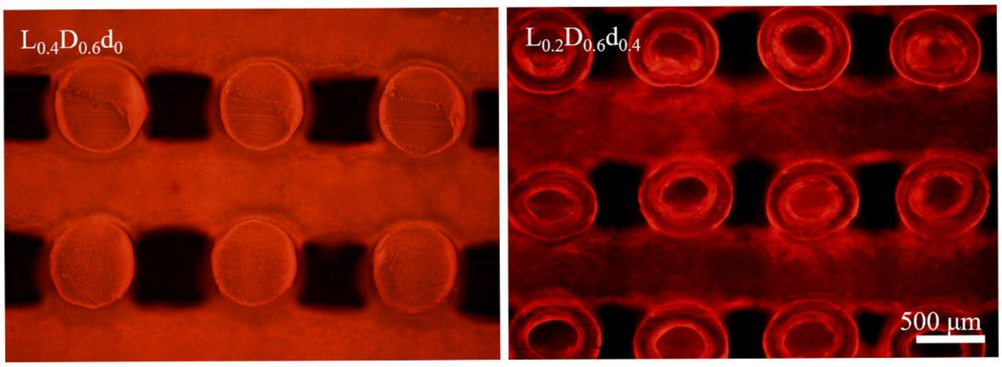

Supplementary Fig. 6 The fluorescent images of hollow structures of $L_{0.4}D_{0.6}d_0$ and $L_{0.2}D_{0.6}d_{0.4}$ HHSs.

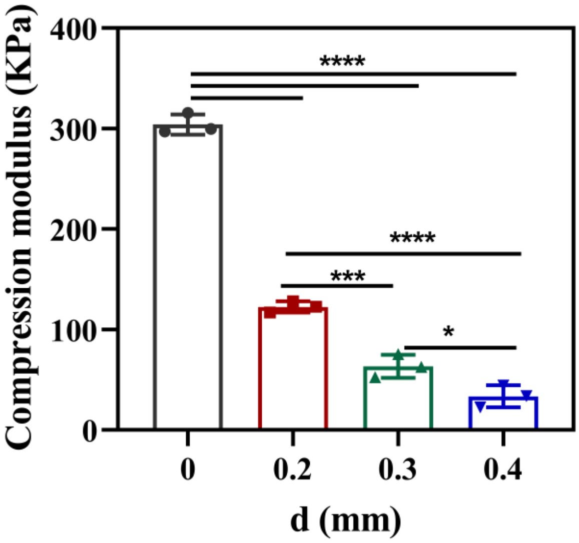

Supplementary Fig. 7 The compression modulus of the $L_{0.4}D_{0.6}d_z$ (z = 0, 0.2, 0.3, and 0.4) HHSs. Dates are presented as means ± s.d, n = 4 per group, statistical significance was calculated using one-way ANOVA method with Tukey's multiple comparisons tests, *P = 0.0234, ***P = 0.0004, ****P < 0.0001. Source data are provided as a Source Data file.

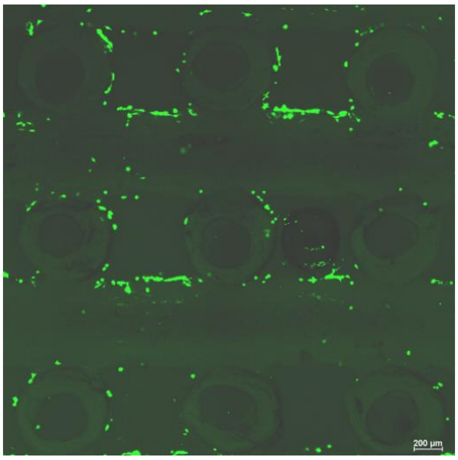

Supplementary Fig. 8 Fluorescence image of loaded cells in the HHS under static condition at day 3.

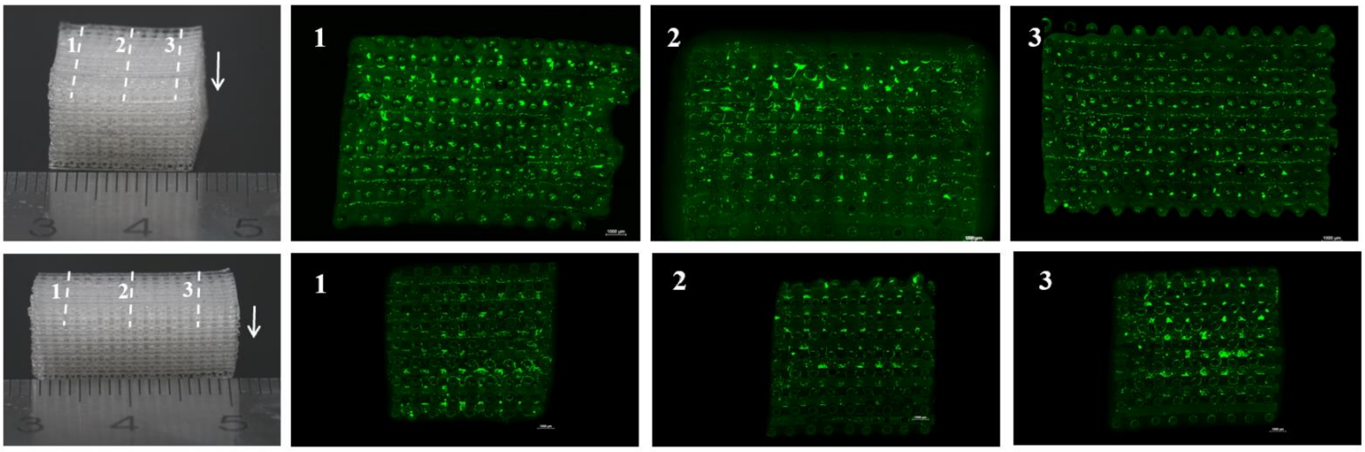

Supplementary Fig. 9 Cell loading to larger size of HHSs (15 ×15 ×10 mm and 20 ×10 ×10 mm) at various crosssections with mechanical responsiveness. Each experiment was repeated three times independently with similar results.

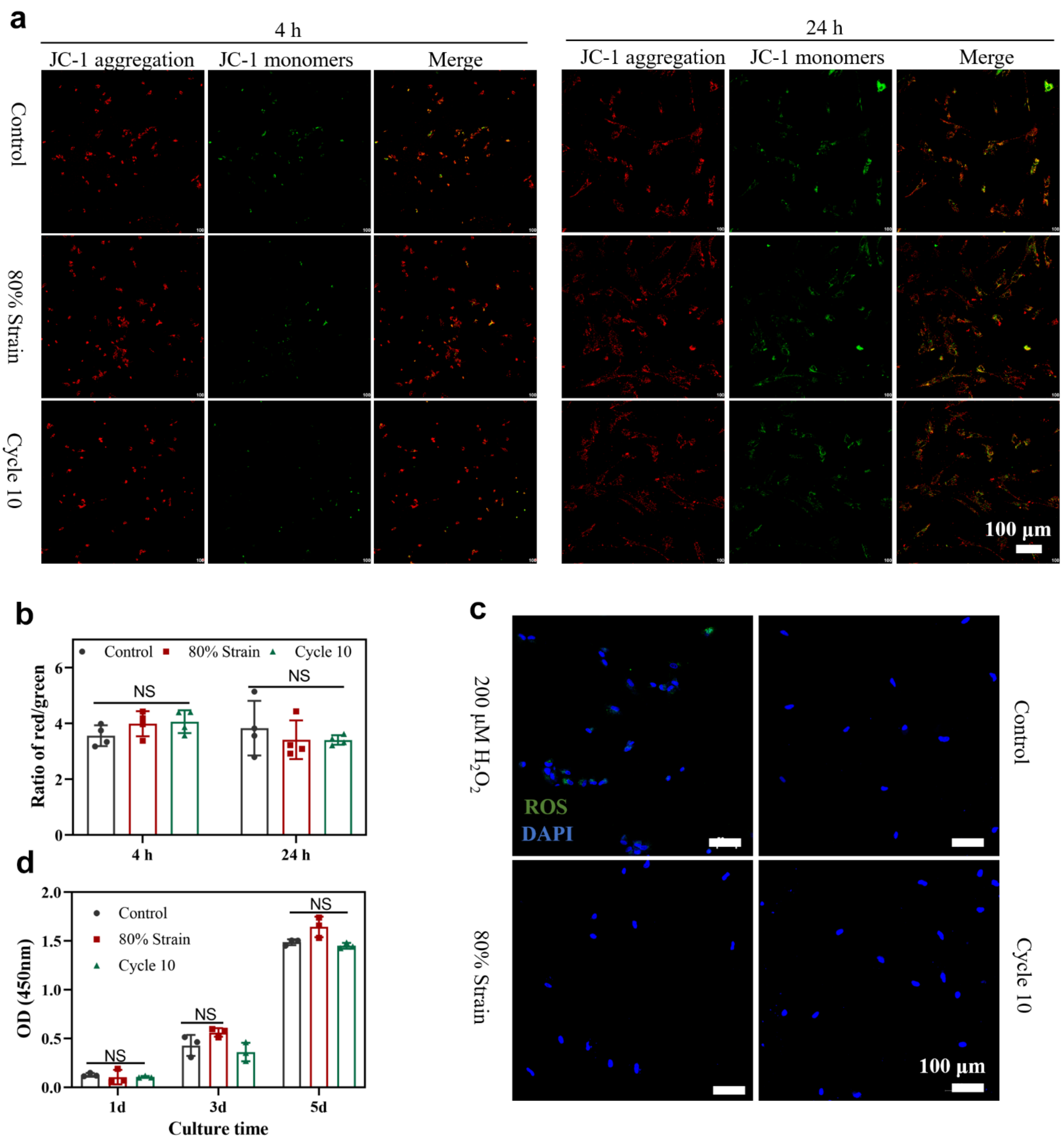

Supplementary Fig. 10 Cell viability after mechanical stimulation with 80% strain and 10 cycles of compression-recovery under 40% strain. a,**b**, The fluorescence images (**a**) and quantitative analysis of the fluorescent intensity of red/green ratio (**b**) of cells stained by JC-1 after undergoing mechanical stimulation for 4 h and 24 h of culture, JC-1 aggregation (red) and JC-1 monomers (green) of cells, the cells that did not undergo mechanical stimulation were as a control group, n = 4. **c**, Fluorescence images of ROS generated by cells via a probe DCFH-DA after undergoing mechanical stimulation for 4 h of culture, the cells cultured with 200 μM H_2O_2 were as a positive group. **d**, CCK8 assay of the cells undergoing mechanical stimulation for 1, 3, and 5 days of culture, n = 3 per group. Dates are presented as means ±s.d, n = 4 per group, statistical significance was calculated using one-way ANOVA method with Tukey's multiple comparisons tests, and NS represents no significant difference ($P > 0.05$) .Each experiment in **a** and **c** was repeated three times independently with similar results. Source data are provided as a Source Data file.

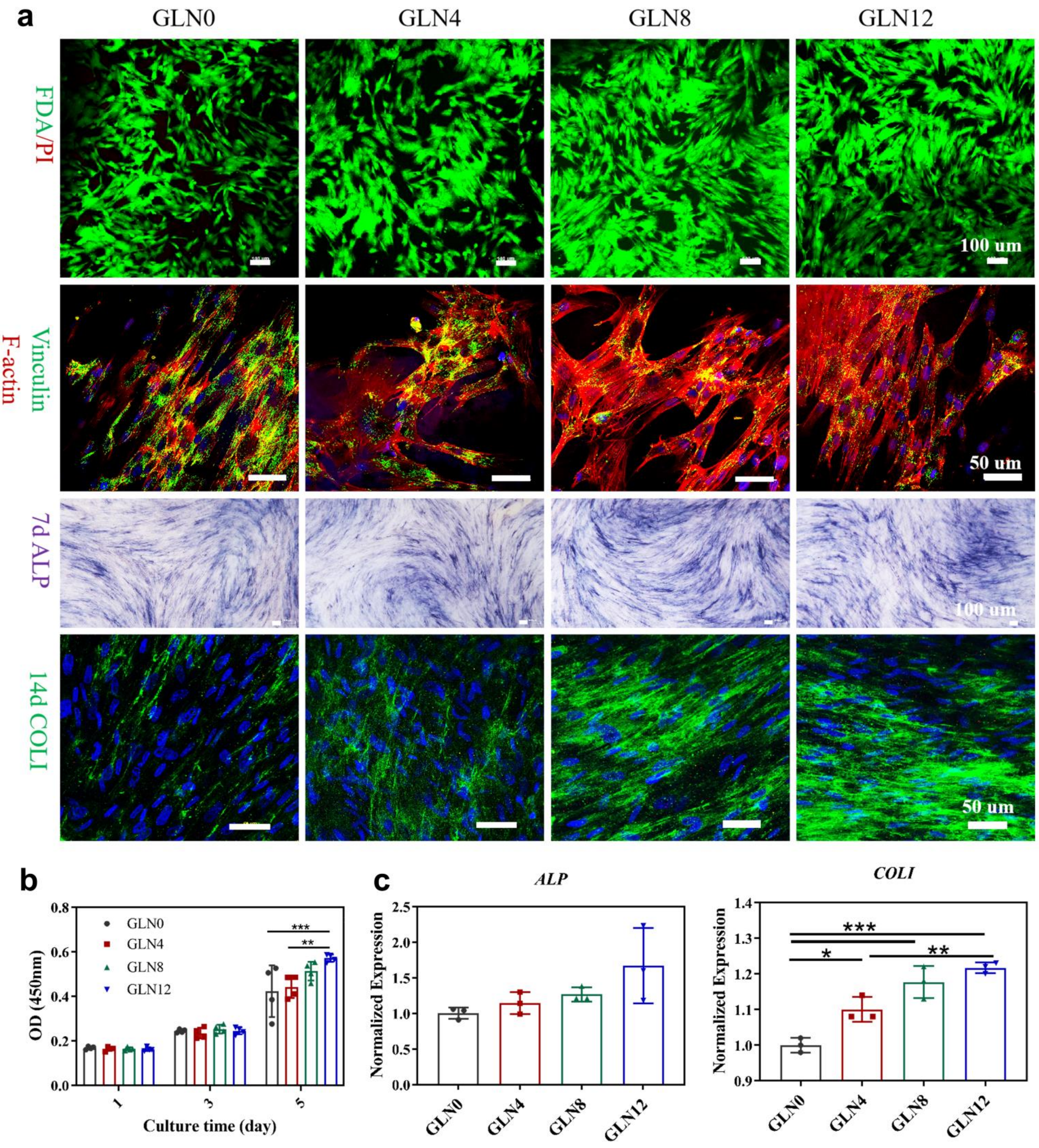

Supplementary Fig. 11 The viability, spreading, proliferation, and osteogenic differentiation of hBMSCs on GLN hydrogels. a, Live (green)/dead (red) staining by FDA and PI at 1 day, vinculin (green) and phalloidin (red) for F-actin at 1 day, ALP staining at 7 days and immunofluorescence staining of Col I at 14 days, each experiment was repeated three times independently with similar results. **b**, CCK8 assay for quantitative analysis of hBMSCs proliferation on GLN hydrogels at 1, 3, and 5 days, $n = 4$, $^{**}P = 0.0034$, $^{***}P = 0.0005$. **c**, Genes expression of ALP and COLI of hBMSCs on GLN hydrogels at 7 days, $n = 3$, *COLI* : $^{*}P = 0.0034$, $^{**}P = 0.0076$, $^{***}P = 0.0005$ (GLN0 and GLN8), $^{***}P = 0.0001$ (GLN0 and GLN8). Dates are presented as means ± s.d, statistical significance was calculated using one-way ANOVA method with Tukey's multiple comparisons tests. Source data are provided as a Source Data file.

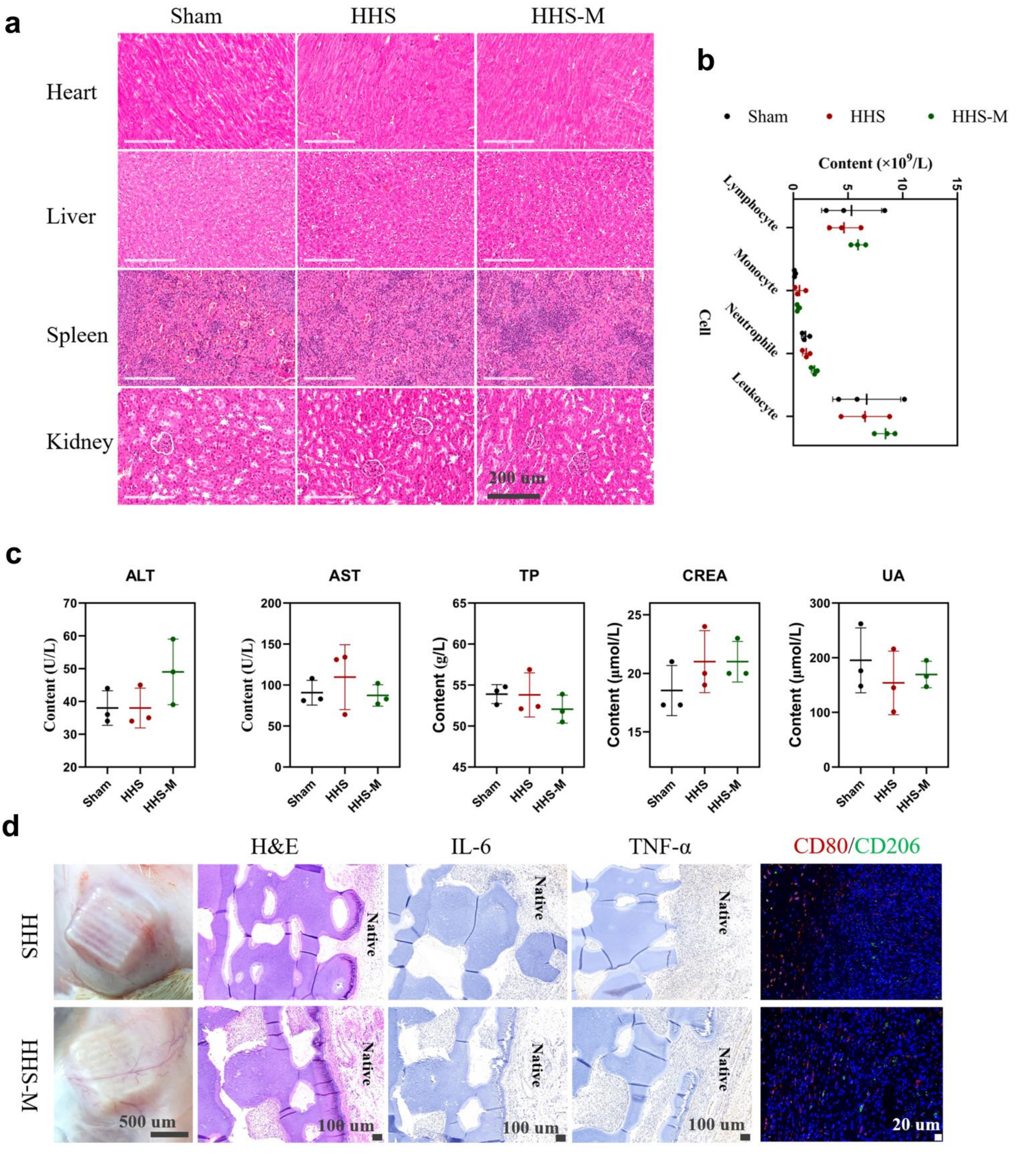

Supplementary Fig. 12 The inflammatory and foreign body responses of HHSs-M for *rat* subcutaneous implantation at 7 days. **a**, H&E staining of the heart, liver, spleen, and kidney. **b**, The complete blood count. **c**, The blood biochemistry analysis, liver function indicators (AST, ALT and TP) and kidney function indicators (CREA and UA). Dates are presented as means ± s.d, *n* = 3 per group. **d**, Photographs of macroscopic implants, H&E staining, immunohistochemical staing of IL-6, TNF-α, CD206 and CD80, each experiment was repeated three times independently with similar results. Source data are provided as a Source Data file.

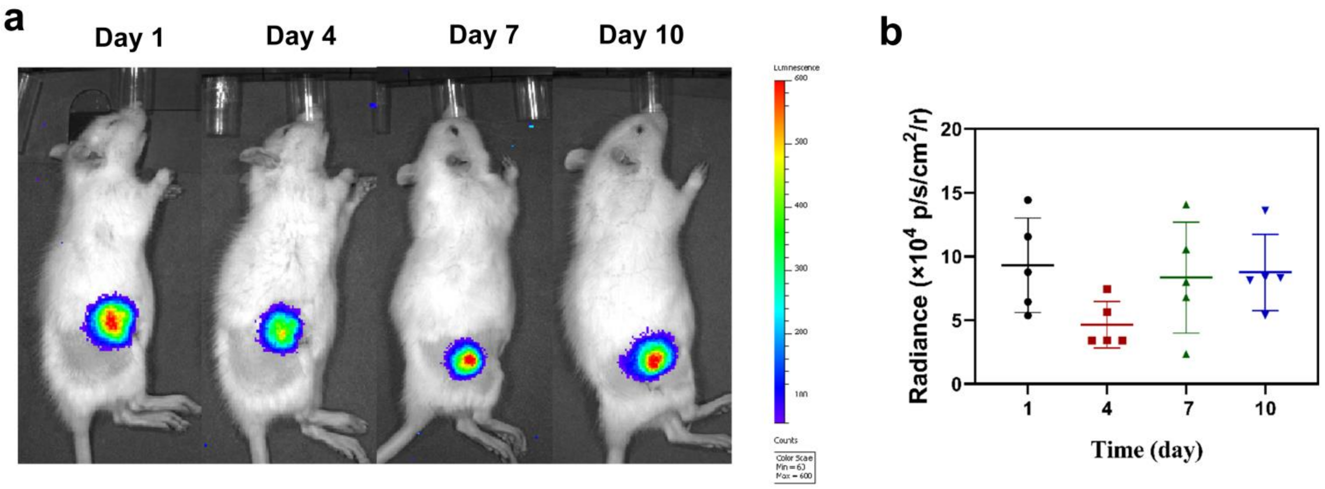

Supplementary Fig. 13 The survival of seeded cells in the HHSs implantation. a, Representative bioluminescence images of *rats* after subcutaneous implantation HHS-M with luciferase over-expressed rBMSCs. **b**, Quantitative analysis of bioluminescence in rats at different time points. Dates are presented as means ± s.d, $n = 5$ per group. Source data are provided as a Source Data file.

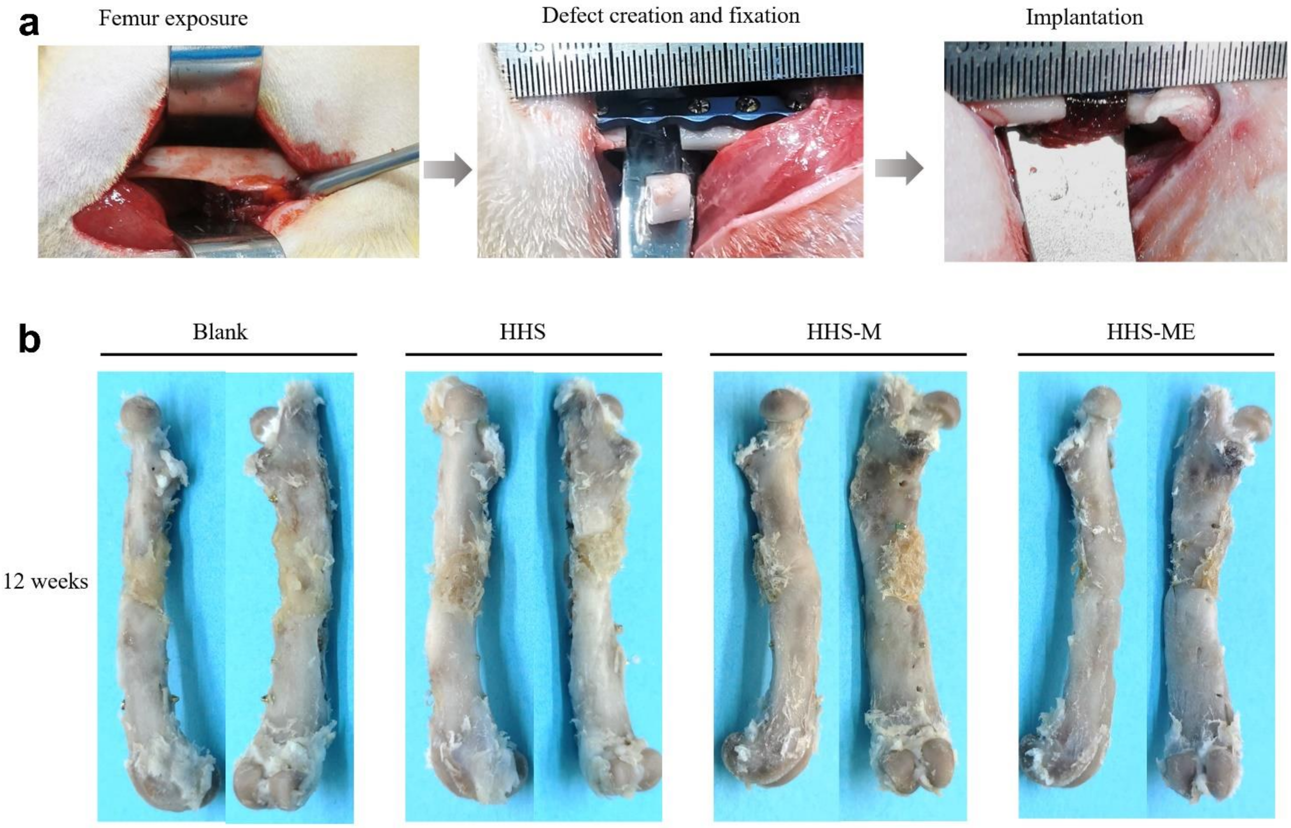

Supplementary Fig. 14 The bone regeneration of the large-size segmental bone defects by the HHS-cells (HHS-M and HHS-ME). a, Photographs of the surgical process. **b**, Photographs of gross view of the femurs of *rats* with 5-mm critical femoral mid-diaphyseal defects at 12 weeks after surgery, the defects were treated with four groups (Blank, HHS, HHS-M, and HHS-ME) respectively.

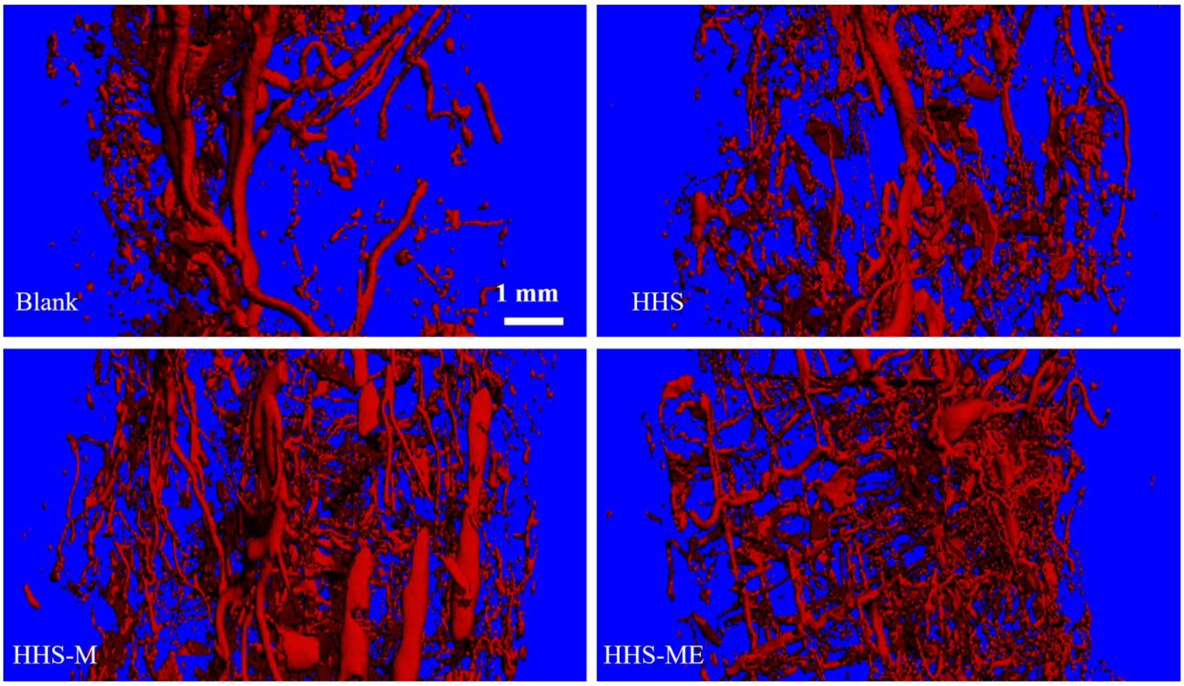

Supplementary Fig. 15 The reconstruction of blood vessels in large-sized segmental bone defects of Blank, HHS, HHS-M, and HHS-ME group at 6 weeks after surgery.

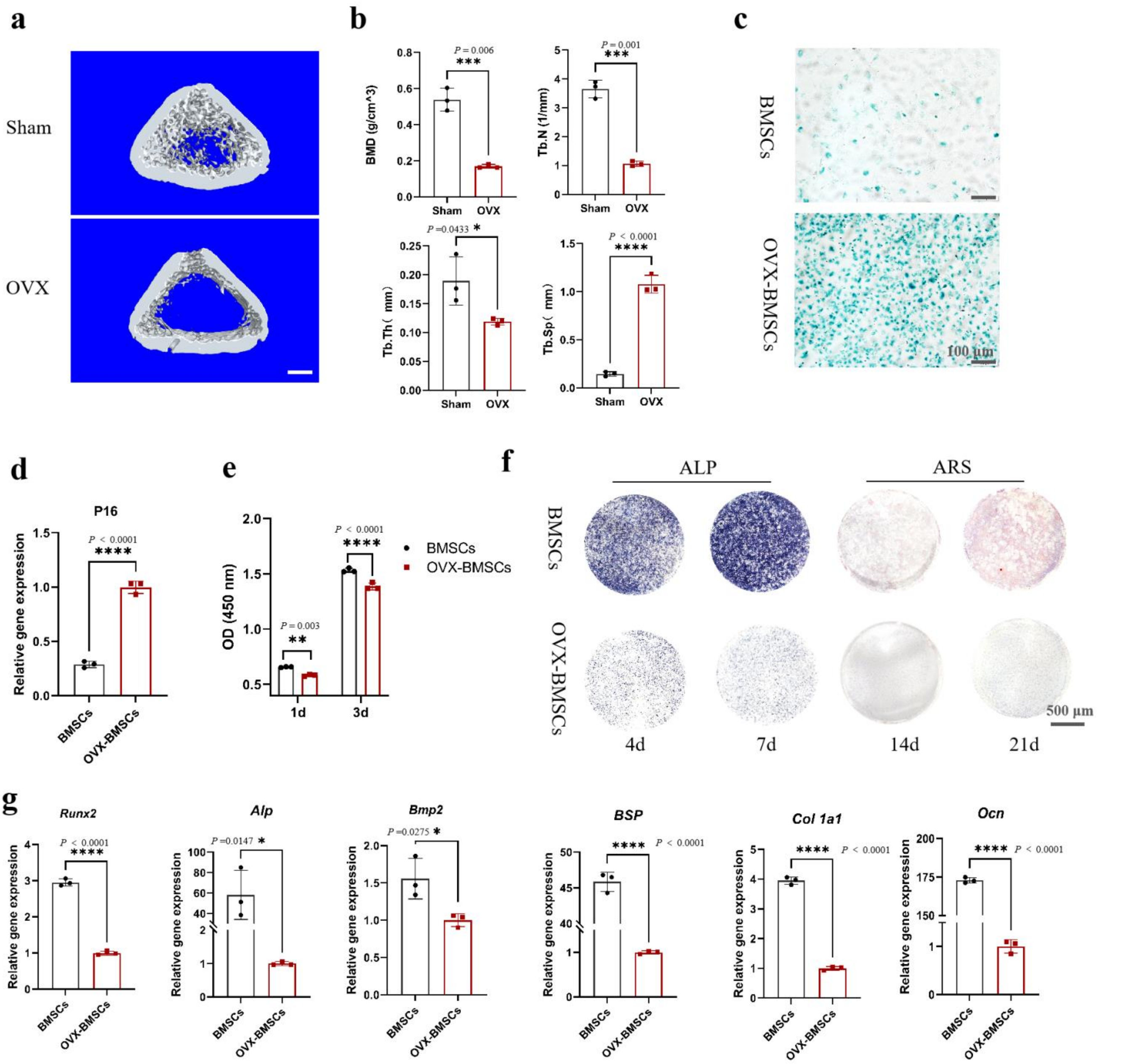

Supplementary Fig. 16 Osteoporotic *rat* models, senescence and osteogenic differentiation of OVX-BMSCs. a, μCT reconstruction images showing cross-section views of sham and ovariectomized groups at week 12 after surgery. **b**, Quantitative analysis of bone mineral density (BMD), bone trabeculae number (Tb.N), bone trabecular thickness (Tb.Th) and trabecular separation (Tb.Sp) from the reconstructed μCT images. **c,d,e,** SA-βgal staining (**c**), Gene expression of *P16* (**d**) and CCK8 assay (**e**) of BMSCs and OVX-BMSCs. **f**, ALP staining at day 4 and 7, and ARS staining at day 14 and 21. **g**, Gene expression of *Runx2*, *Alp*, *Bmp2*, *Bsp*, *Col 1a1* and Ocn on 7 days. Dates are presented as means ± s.d, *n* = 3 per group, statistical significance was calculated using Student's t tests. Source data are provided as a Source Data file.

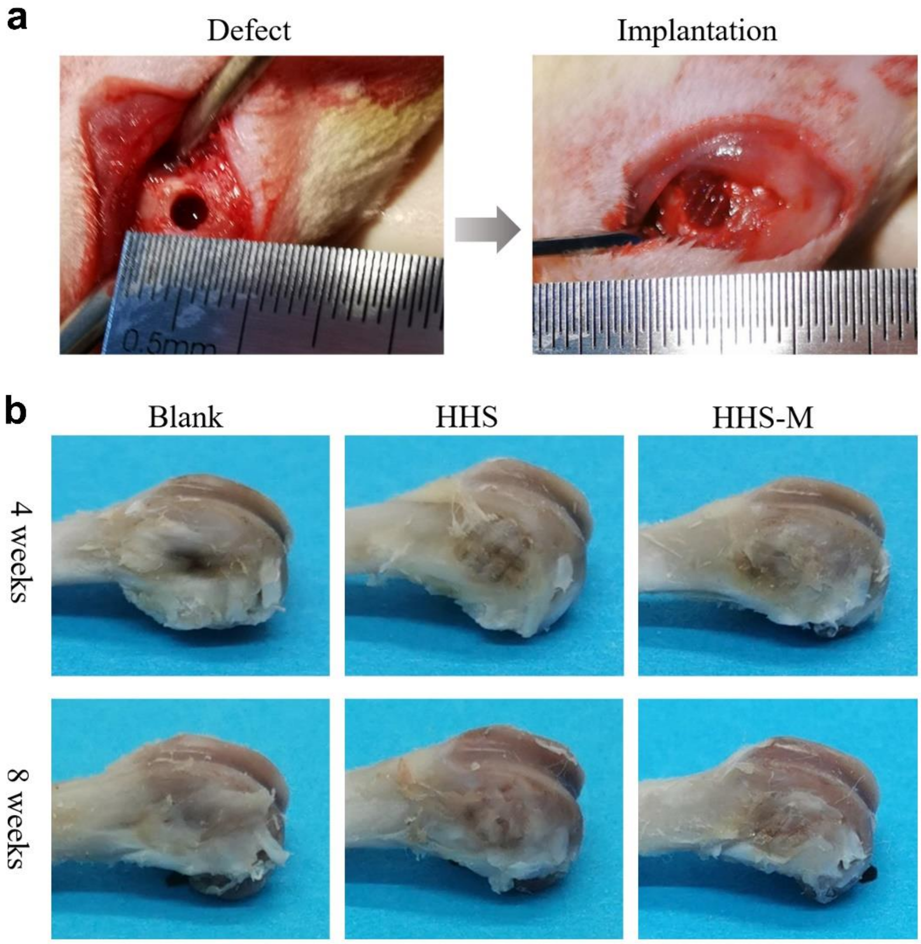

Supplementary Fig. 17 The bone regeneration of osteoporotic bone defects by the HHS-cells (HHS-M). a, Photographs of the surgical process. **b**, Photographs of the gross view of distal femurs of *rats* with critical defects (φ3 mm × h 4 mm) at 4 and 8 weeks after surgery, the defects were treated with three groups (Blank, HHS, and HHS-M) respectively.

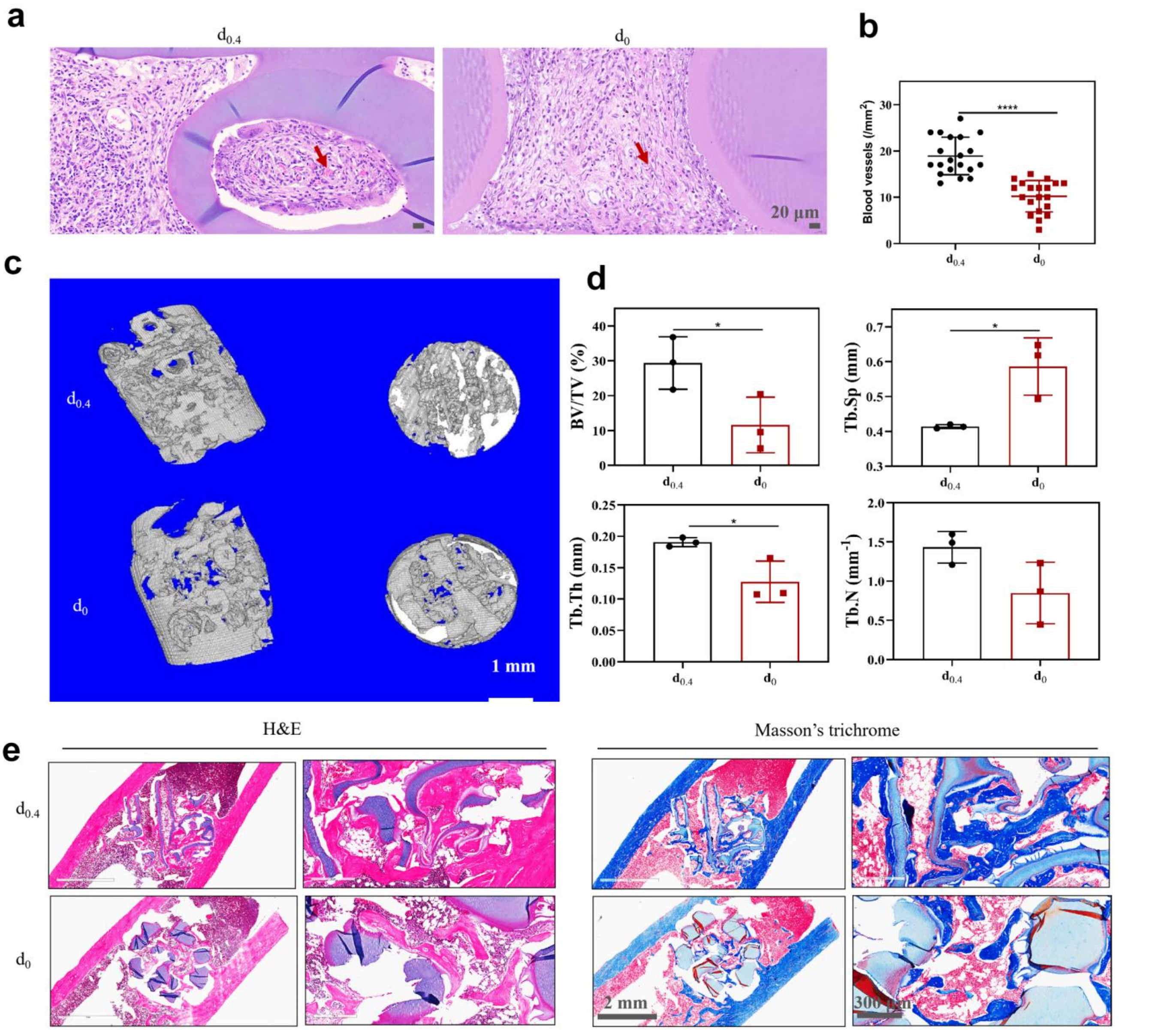

Supplementary Fig. 18 The *rat* subcutaneous implantation and repair of distal femoral metaphyseal defects to evaluate the effect of hollow structure of HHSs ($L_{0.4}D_{0.6}d_0$ and $L_{0.4}D_{0.6}d_{0.4}$) on blood vessels ingrowth and bone formation. a, H&E staining of the subcutaneous implantation samples at 2 weeks, red arrows represent blood vessels that grew in the HHSs after surgery. **b**, Quantitative analysis of the blood vessels that grew in the HHSs according to the H&E staining, $n = 4$, 5 images for each sample, ****$P < 0.0001$. **c**, μCT reconstruction images of new bone view of metaphyseal bone in the defective region at 8 weeks after surgery. **d**, Quantitative analysis of bone volume to the total defect volume (BV/TV) *$P = 0.0488$, bone trabeculae number (Tb.N) $P = 0.0840$, trabecular separation (Tb.Sp) *$P = 0.0225$, and trabecular thickness (Tb.Th) *$P = 0.0317$ from the reconstructed μCT images at 8 weeks. Dates are presented as means ± s.d, statistical significance was calculated using Student's t-tests, $n = 3$. **e**, H&E and Masson's trichrome staining at 8 weeks, obviously new bone formation in the d_0 HHS group. Each experiment in **a, c** and **e** was repeated three times independently with similar results. Source data are provided as a Source Data file.

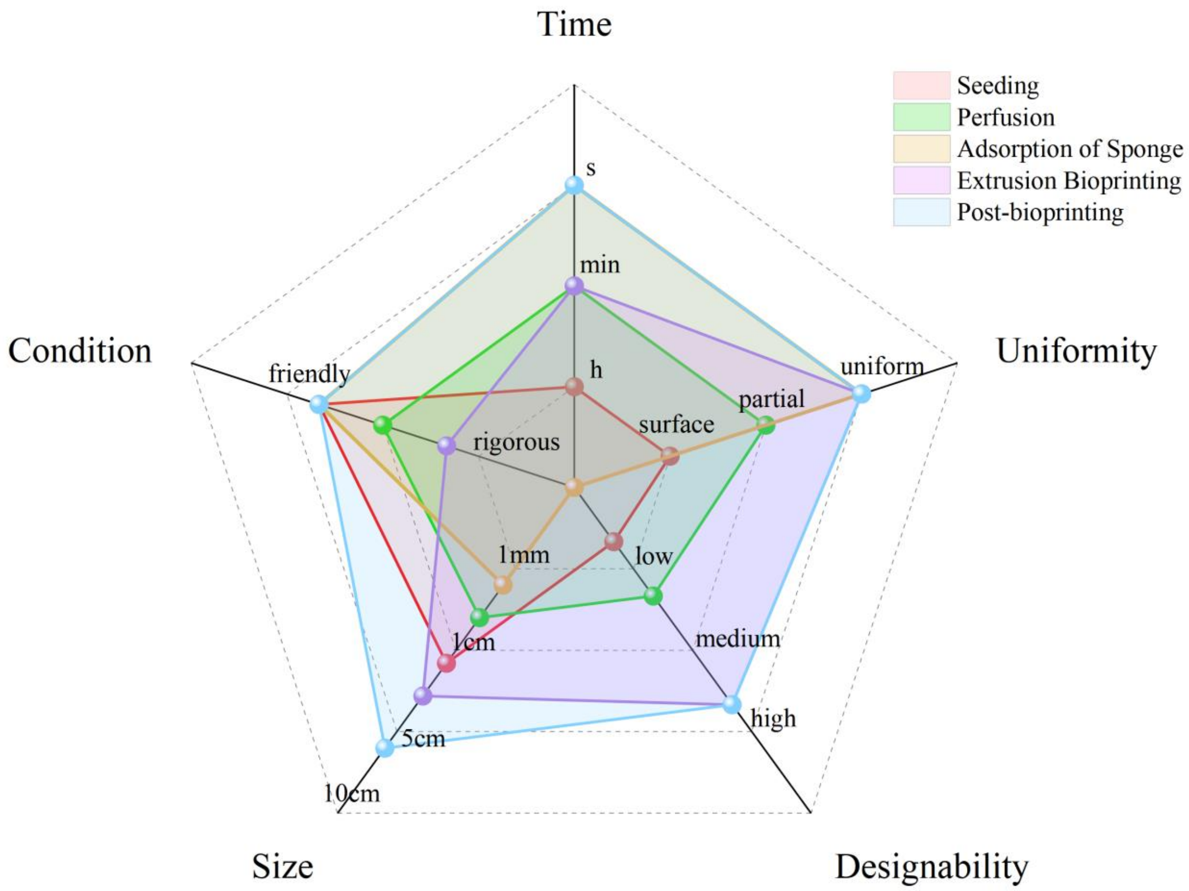

Supplementary Fig. 19 The comparative analysis on the advantages of the Post-bioprinting among different cell loading methods.

Supplementary Tables

Supplementary Table 1. The ratio of theoretical volume of V_1 and V_2 to the total volume of HHSs and water uptake ratio of HHSs in static condition

Group	Ratio of volume of V_1 (%)	Ratio of volume of V_2 (%)	Water uptake ratio (%)
$L_{0.4}D_{0.6}d_{0.4}$	22.1±3.3	39.2±5.8	33.7±1.4
$L_{0.4}D_{0.6}d_{0.3}$	12.7±1.9	39.2±5.8	38.3±1.8
$L_{0.4}D_{0.6}d_{0.2}$	5.7±1.0	39.2±5.8	34.4±3.7
$L_{0.4}D_{0.6}d_{0}$	0	39.2±5.8	37.9±2.8
$L_{0.2}D_{0.6}d_{0.4}$	28.7±2.5	27.7±5.4	27.6±0.5
$L_{0.6}D_{0.6}d_{0.4}$	18.3±2.8	49.0±4.3	49.0±0.5

Supplementary Table 2. The forward and reverse primer sequences for qRT-PCR

Gene	Forward primer sequences (5’-3’)	Reverse primer sequence (5’-3’)
GAPDH	TCAAGGCTGAGAACGGGAA	TGGGTGGCAGTGATGGCA
ALP	TGCAGGATCGGAACGTCAAT	GAGTTGGTAAGGCAGGGTCC
Col I	CACACGTCTCGGTCATGGTA	AAGAGGAAGGCCAAGTCGAG
BMP2	GCATCGCGCCCCTTATCC	TTCCTCGATGGCTTCTTCGT
BSP	CCAGCCAGGACTGCCGAAGG	CGCTGCCTCCCTGGACTGGA
Runx2	AGATGGGACTGTGGTTACCG	GGACCGTCCACTGTCACTTT
OCN	CCGTTTAGGGCATGTGTTGC	CCGTCCATACTTTCGAGGCA
P16	GTACCCATACAGGTGATGA	GGTGCAGTACTACCAGAGTGT
