# Supplementary material for: A mechanical-assisted post-bioprinting strategy for challenging bone defects repair
Source: Nat Commun. 2024 Apr 26;15:3565. doi: 10.1038/s41467-024-48023-8 (PMC11053166; doi:10.1038/s41467-024-48023-8)
Supplement: Supplementary file 3 — Description of Additional Supplementary Files [file 41467_2024_48023_MOESM3_ESM.pdf]

Description of Additional Supplementary Files

Supplementary Movie 1 Actual process of printing bone-shaped construct

Supplementary Movie 2 Compression tests of HHSs with 80% stain

Supplementary Movie 3 Visual process of compression-recovery of HHSs

Supplementary Movie 4 Processes of two cell seeding approaches: direct cell seeding and cell seeding by V_2-mechanical response

Supplementary Movie 5 Processes of V_1+V_2-mechanical response
