# Supplementary material for: A mechanical-assisted post-bioprinting strategy for challenging bone defects repair
Source: Nat Commun. 2024 Apr 26;15:3565. doi: 10.1038/s41467-024-48023-8 (PMC11053166; doi:10.1038/s41467-024-48023-8)
Supplement: Supplementary file 9 — Reporting Summary [file 41467_2024_48023_MOESM9_ESM.pdf]

nature portfolio

Corresponding author(s): Changshun Ruan

Last updated by author(s): Apr 15, 2024

Reporting Summary

Nature Portfolio wishes to improve the reproducibility of the work that we publish. This form provides structure for consistency and transparency in reporting. For further information on Nature Portfolio policies, see our Editorial Policies and the Editorial Policy Checklist.

Statistics

For all statistical analyses, confirm that the following items are present in the figure legend, table legend, main text, or Methods section.

n/a	Confirmed	
☐	☒	The exact sample size (*n*) for each experimental group/condition, given as a discrete number and unit of measurement
☐	☒	A statement on whether measurements were taken from distinct samples or whether the same sample was measured repeatedly
☐	☒	The statistical test(s) used AND whether they are one- or two-sided<br>*Only common tests should be described solely by name; describe more complex techniques in the Methods section.*
☒	☐	A description of all covariates tested
☒	☐	A description of any assumptions or corrections, such as tests of normality and adjustment for multiple comparisons
☐	☒	A full description of the statistical parameters including central tendency (e.g. means) or other basic estimates (e.g. regression coefficient) AND variation (e.g. standard deviation) or associated estimates of uncertainty (e.g. confidence intervals)
☐	☒	For null hypothesis testing, the test statistic (e.g. F, t, r) with confidence intervals, effect sizes, degrees of freedom and P value noted<br>*Give P values as exact values whenever suitable.*
☒	☐	For Bayesian analysis, information on the choice of priors and Markov chain Monte Carlo settings
☒	☐	For hierarchical and complex designs, identification of the appropriate level for tests and full reporting of outcomes
☒	☐	Estimates of effect sizes (e.g. Cohen's *d*, Pearson's *r*), indicating how they were calculated

Our web collection on statistics for biologists contains articles on many of the points above.

Software and code

Policy information about availability of computer code

Data collection	Absorbance data were collected from microplate reader (Thermo Scientific, Multiskan GO, USA).<br>Fluorescence image data of cells were collected from confocal laser scanning microscopy (Leica SP8, Germany; Nikon C2, Japan)<br>Gross images were collected form a digital camara (Sony, a7m3, Japan)<br>The rheological data were collected using a shear rheometer (MCR 302, Anton Paar, Austria)<br>The microstructure of hydrogels were obtained from a scanning electron microscopy (SEM, Hitachi S-4800, Japan)<br>The elemental analysis was detected using an energy dispersive spectrophotometer (EDS, Hitachi, Japan).<br>Mechanical data was collected from a mechanical analyzer (Care, IBTC-300SL, China)<br>Fluorescence image of HHS and Fluorescence image of cells were IVIS imaging systems (Caliper Life Sciences, USA)
Data analysis	Microscopic images were analyzed by using ImageJ.<br>Statistical data analyses were performed using GraphPadPrism 8.<br>µCT data were analyses by Bruckers software and Scanco software.

For manuscripts utilizing custom algorithms or software that are central to the research but not yet described in published literature, software must be made available to editors and reviewers. We strongly encourage code deposition in a community repository (e.g. GitHub). See the Nature Portfolio guidelines for submitting code & software for further information.

Data

Policy information about availability of data

All manuscripts must include a data availability statement. This statement should provide the following information, where applicable:

- Accession codes, unique identifiers, or web links for publicly available datasets
- A description of any restrictions on data availability
- For clinical datasets or third party data, please ensure that the statement adheres to our policy

The main data supporting the results in this study are available within the paper and its Supplementary Information and Movies. All data generated in this study are provided as source files.

Research involving human participants, their data, or biological material

Policy information about studies with human participants or human data. See also policy information about sex, gender (identity/presentation), and sexual orientation and race, ethnicity and racism.

Reporting on sex and gender	n/a
Reporting on race, ethnicity, or other socially relevant groupings	n/a
Population characteristics	n/a
Recruitment	n/a
Ethics oversight	n/a

Note that full information on the approval of the study protocol must also be provided in the manuscript.

Field-specific reporting

Please select the one below that is the best fit for your research. If you are not sure, read the appropriate sections before making your selection.

☒ Life sciences ☐ Behavioural & social sciences ☐ Ecological, evolutionary & environmental sciences

For a reference copy of the document with all sections, see nature.com/documents/nr-reporting-summary-flat.pdf

Life sciences study design

All studies must disclose on these points even when the disclosure is negative.

Sample size	n =10 independent samples for Figure 2d,<br>n =3 independent samples for Figure 4b, 4c, 4e, 4f, 4h, 4i,<br>n =4 independent samples for Figure 4a, 4d,<br>n =5 biologically independent samples for Figure 5b, 5ciii, 5eiii, 5eiv,<br>n =8 biologically independent samples for Figure 5diii,<br>n =4 biologically independent samples for Figure 6d,<br>n =3 biologically independent samples for Figure 7c of blank group,<br>n =4 biologically independent samples for Figure 7c of HHS and HHS-M group.
Data exclusions	No data was excluded from the analyses
Replication	We performed each experiment for a minimum of three times in a month.
Randomization	For in vivo and in vitro studies, samples were randomly allocated to different experimental groups.
Blinding	Both in vitro cells and in vivo rat allocations were conducted in a blinded manner.

Reporting for specific materials, systems and methods

We require information from authors about some types of materials, experimental systems and methods used in many studies. Here, indicate whether each material, system or method listed is relevant to your study. If you are not sure if a list item applies to your research, read the appropriate section before selecting a response.

Materials & experimental systems

n/a	Involved in the study
☐	☒ Antibodies
☐	☒ Eukaryotic cell lines
☒	☐ Palaeontology and archaeology
☐	☒ Animals and other organisms
☒	☐ Clinical data
☒	☐ Dual use research of concern
☒	☐ Plants

Methods

n/a	Involved in the study
☒	☐ ChIP-seq
☒	☐ Flow cytometry
☒	☐ MRI-based neuroimaging

Antibodies

Antibodies used

For in vitro immunofluorescence studies, following antibodies were used:
Primary antibodies: CD31 (ab28364, Abcam), VWF (SC-365712, Santa), vinculin (ab129002, Abcam), ColI (ab34710,Abcam).
Secondary antibodies: goat-anti-rabbit IgG Alexa Fluor 488 (ab150077, Abcam), goat-anti-mouse IgG Alexa Fluor 647 (ab150115, Abcam).

For in vivo immunofluorescence studies, following antibodies were used:
Primary antibodies: IL6 (ab9324, Abcam), TNF-α (ab220210, Abcam), CD80 antibody (A001961, Boster), CD206 (AB64693, Abcam)
Secondary antibodies: Goat Anti-Mouse IgG H&L (ab205719, Abcam), goat-anti-rabbit IgG Alexa Fluor 488 (ab150077, Abcam), goat-anti-rabbit IgG Alexa Fluor 647 (ab150083, Abcam)

Validation

All primary antibodies have been validated for the species and application by the manufacturer's website and relevant references.
CD31(Abcam, ab28364, 1/200 for IF), was validated by immunofluorescence staining of human cells, validation information can be found on the manufacturer's website.
VWF (SC-365712, Santa, 1/200 for IF), was validated by immunofluorescence staining of human cells, validation information can be found on the manufacturer's websites.
Vinculin (Abcam, ab129002, 1/100 for IF), was validated by immunofluorescence staining of human cells, validation information can be found on the manufacturer's.
ColI (Abcam, ab34710, 1/200 for IF), was validated by immunofluorescence staining of human cells, validation information can be found on the manufacturer's website.
IL6 (Abcam, ab9324, 1/200 for immunohistochemistry), was validated by immunohistochemistry staining of rat tissue, validation information can be found on the manufacturer's website .
TNF-α (Abcam, ab220210, 1/200 for immunohistochemistry), was validated by iimmunohistochemistry staining of rat tissue, validation information can be found on the manufacturer's website.
CD80 antibody (Boster, A001961, 1/200 for IF), was validated by immunofluorescence staining of rat tissue, validation information can be found on the manufacturer's website .
CD206 (Abcam, AB64693, 1/200 for IF), was validated by immunofluorescence staining of rat tissue, validation information can be found on the manufacturer's website.
Goat-anti-rabbit IgG Alexa Fluor 488 (ab150077, Abcam, 1/200 for IF), was validated by immunofluorescence staining of human cells and rat tissue, validation information can be found on the manufacturer's website.
Goat-anti-mouse IgG Alexa Fluor 647 (ab150115, Abcam, 1/200 for IF), was validated by immunofluorescence staining of human cells, validation information can be found on the manufacturer's website.
Goat-anti-rabbit IgG Alexa Fluor 647 (ab150083, Abcam, 1/200 for IF), was validated by immunofluorescence staining of rat tissue, validation information can be found on the manufacturer's website.
Goat Anti-Mouse IgG H&L (ab205719, Abcam, 1/2000 for immunohistochemistry), was validated by iimmunohistochemistry staining of rat tissue, validation information can be found on the manufacturer's website .

Eukaryotic cell lines

Policy information about cell lines and Sex and Gender in Research

Cell line source(s)	We did not use cell linehave, we used primary cells and have stated the source in the manuscript
Authentication	n/a
Mycoplasma contamination	All primary cells tested negative for mycoplasma contamination.
Commonly misidentified lines (See ICLAC register)	We didn't use any cell lines in this study, so no misidentified cell lines

Animals and other research organisms

Policy information about studies involving animals; ARRIVE guidelines recommended for reporting animal research, and Sex and Gender in Research

Laboratory animals	Male and female SD rats (12 weeks old) were purchased from Guangzhou Vital River Laboratory Animal Technology Co., Ltd. and kept under a 12/12h light/dark cycle, 24–26 °C, and 60% humidity.<br>Anesthesia was performed with 5% isoflurane and O2. Animals were placed on a 37 °C warm heating pad, isoflurane was lowered to 2 to 2.5% to maintain anesthesia during the entire surgery. After wound closure, animals were injected with 8 U/mL penicillin and transferred back into their cage. To relieve the pain, all rats were orally administrated with ibuprofen (10mg/kg, PHR1004, Merck) in drinking water for 24h after surgery. Animals were euthanized by CO2 asphyxiation at the end of the experiment.
Wild animals	The study didn't involve wild animals.
Reporting on sex	For the rat femoral segmental defect experiment, the information of sex has not been collected. For the rat osteoporotic bone defect experiment, female rats were used.
Field-collected samples	The study did not involve samples collected from the field.
Ethics oversight	The animal experiments were approved by the Institutional Animal Care and Use<br>Committee of Shenzhen Institute of Advanced Technology, Chinese Academy of Science.

Note that full information on the approval of the study protocol must also be provided in the manuscript.

Plants

Seed stocks	n/a
Novel plant genotypes	n/a
Authentication	n/a
